# Maintained Symptom Control Following a Shortage-Driven Switch to Immediate-Release Dextroamphetamine: A Case Report

**DOI:** 10.7759/cureus.110860

**Published:** 2026-06-14

**Authors:** Daniel Singer, Priti M Kothari

**Affiliations:** 1 College of Liberal Arts and Sciences, University of Florida, Gainesville, USA; 2 Department of Psychiatry, Priti Kothari MD PA, Boca Raton, USA; 3 College of Nursing, University of Florida, Gainesville, USA

**Keywords:** adult attention-deficit/hyperactivity disorder (adhd), amphetamine, cns psychostimulants, dextroamphetamine, stimulant shortage

## Abstract

ADHD is a neurodevelopmental condition marked by persistent symptoms of inattention, hyperactivity, and impulsivity. The gold standard pharmacotherapy for the treatment of ADHD is CNS psychostimulants. However, a shortage of CNS psychostimulants used for the treatment of ADHD has persisted since 2022, and many patients have been forced to switch medications or have been unable to obtain medication at all, raising concerns about symptom management and quality of life. This case report describes a 30-year-old female who was unable to obtain lisdexamfetamine amid the shortage, found mixed amphetamine salts ineffective, but found success with immediate-release dextroamphetamine sulfate, a less frequently used amphetamine. This report serves to demonstrate that immediate-release dextroamphetamine sulfate may be a viable alternative stimulant to address the shortage; however, more research must be conducted to confirm these findings.

## Introduction

ADHD is a developmental disorder characterized by chronic symptoms of inattention, hyperactivity, and impulsivity. The most effective treatment for ADHD is widely known to be CNS psychostimulants, mainly amphetamine or methylphenidate [1]. Due to the shortage of ADHD stimulant medication beginning in 2022, some patients have had significant difficulty obtaining their stimulant medication [2]. This leads to treatment gaps and untreated ADHD, which can have damaging implications. A 2024 study interviewed patients and found that many experienced a relapse of debilitating ADHD symptoms and subsequently experienced detrimental effects on their professional, educational, and personal lives. The study also reported that patients experienced driving long distances for medication, receiving partial fills, and switching medications [3]. This essentially requires unmedicated ADHD patients with impaired inattention and working memory to put both to work in order to find their medication; the results of the harm caused by the shortage warrant seeking methods to mitigate the effects of the shortage. One notable option requires transitioning patients to another formulation or molecule. This case report describes a successful outcome in symptom control for a patient who utilized this strategy.

## Case presentation

We report the case of a 30-year-old woman who presented to our clinic with a chief complaint of irritability and inattention while on her current ADHD medication. She reported that her prior psychiatric provider had referred her to our clinic due to retirement. The patient expressed interest in switching to an alternative medication that would adequately control her symptoms. Upon evaluation, she was taking mixed amphetamine salts (MAS) extended-release (XR) 20 mg every morning (QAM), following a prior medication change related to stimulant shortages.

Since this switch, she reported episodes of increased sensitivity to stressors, anger, frustration, impatience, tachycardia, and diaphoresis. These episodes typically occurred around 3 p.m., lasting approximately two to three hours at peak intensity, with gradual improvement throughout the evening. Symptoms persisted at a lower intensity until bedtime. Despite some improvement, irritability negatively affected her relationships. She also endorsed mild fidgeting, difficulty concentrating, impaired planning, forgetfulness, and task paralysis, which further impacted her academic and interpersonal functioning.

She had no notable general medical history or abnormal physical examination findings. Psychiatric history began in adolescence, when she was diagnosed with generalized anxiety disorder at an unclear age and prescribed a selective serotonin reuptake inhibitor (exact agent unknown). She reported no improvement in symptoms and multiple adverse effects, and the medication was tapered and discontinued.

In college, she was reevaluated and diagnosed with ADHD. She trialed methylphenidate (MPH) immediate release (IR), MPH XR, and dexmethylphenidate XR, all of which failed to adequately control symptoms and were associated with increased emotional reactivity. She subsequently achieved adequate symptom control with either lisdexamfetamine (LDX) 30 mg QAM or MAS IR 20 mg twice daily (BID). However, she was transitioned due to stimulant shortages.

Family history was notable for paternal ADHD and paternal grandparents with alcohol and gambling addiction. She denied a maternal psychiatric history. She also denied any history of psychiatric hospitalization, suicidal ideation or behavior, bipolar disorder, psychosis, trauma, obsessive symptoms, or substance use disorder. She denied current or recent suicidal or homicidal ideation. She was alert and oriented to person, place, time, and situation, with intact judgment and safe functional capacity. She drove herself to the appointment without incident and reported stable housing, no safety concerns, and a supportive social network.

The patient was born full term via an uncomplicated vaginal delivery, with normal developmental milestones. She reported lifelong inattentive and impulsive symptoms predating treatment, though she performed as an above-average student in primary and secondary education. She was a full-time graduate student with a demanding academic workload. She denied any history of physical, sexual, or emotional abuse, neglect, exposure to domestic violence, bullying, or legal issues, including arrests, convictions, or incarceration.

**Figure 1:**
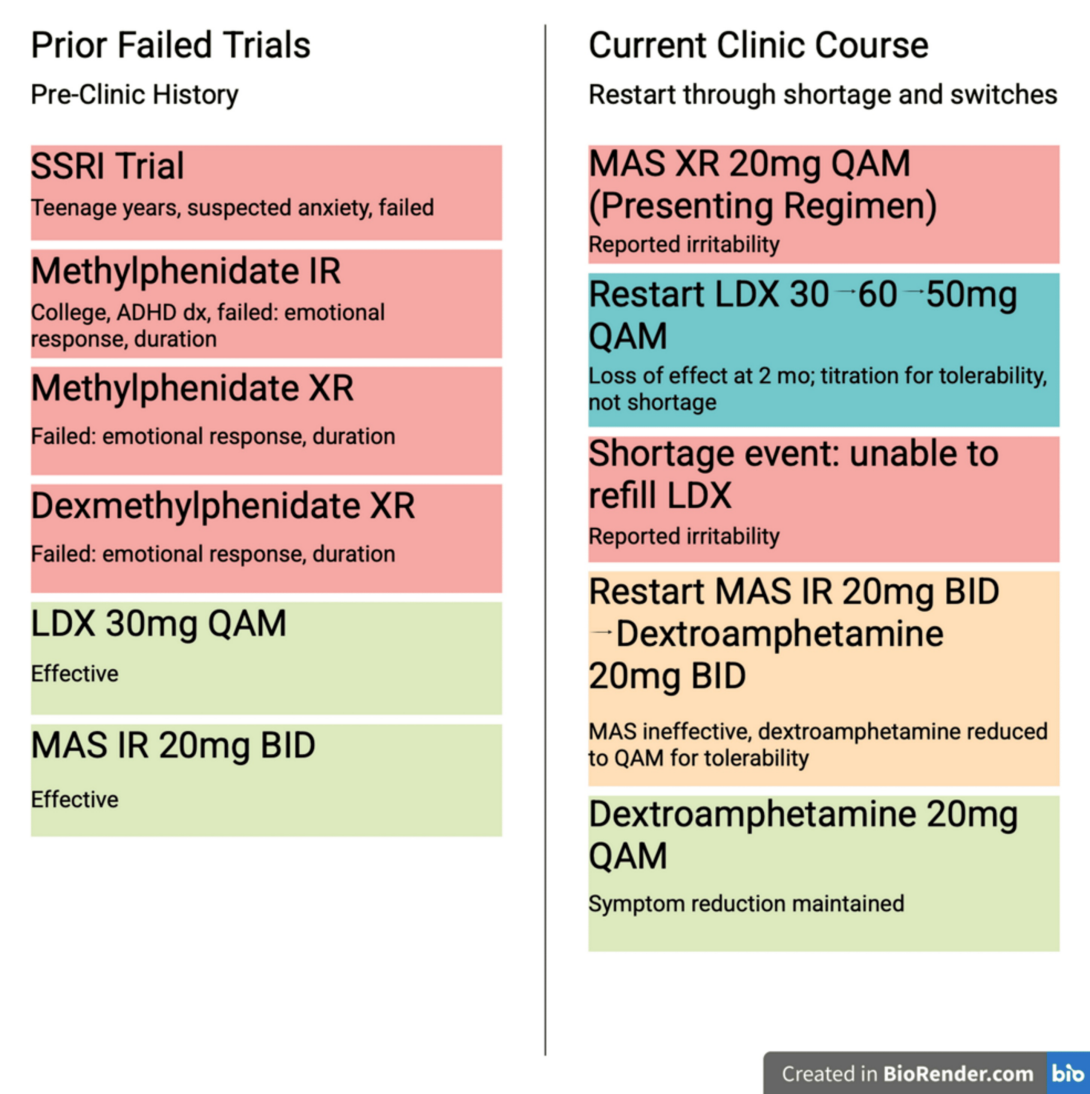
Timeline of psychiatric medication trials, including prior failed trials and current clinic course showing stimulant shortage-driven medication switches BID, twice daily; IR, immediate release; LDX, lisdexamfetamine; MAS, mixed amphetamine salts; QAM, in the morning; SSRI, selective serotonin reuptake inhibitor; XR, extended release This figure was created using BioRender.com (BioRender, Toronto, Canada).

Upon evaluation, she was diagnosed with ADHD, predominantly inattentive type (F90.0). MAS XR 20 mg QAM was discontinued, and she was initiated on LDX 30 mg QAM, titrated to 60 mg QAM, and subsequently adjusted to 50 mg QAM. She was later transitioned to MAS IR 20 mg BID, followed by dextroamphetamine (D-AMPH) IR 20 mg BID, and finally adjusted to D-AMPH IR 20 mg QAM (Figure 1).

## Discussion

Our clinic diagnosed the patient with ADHD, predominantly inattentive (F90.0). According to the DSM-5 criteria for diagnosing ADHD, the predominantly inattentive presentation is met when a patient meets Criterion A, representing inattention, for the past six months, but not Criterion B, which represents hyperactivity [4]. Our clinic discontinued her currently prescribed MAS 20 mg XR QAM due to the lack of efficacy, and our patient reported “episodes,” which we understand to be a response to the offset of action of MAS 20 mg XR [5].

When choosing the adequate medication to trial, we noted our patient had previously achieved successful symptom reduction with both LDX 30 mg QAM and MAS IR 20 mg BID and had previously not responded to any methylphenidate or dexmethylphenidate product. This is supported by evidence that suggests amphetamine-based products are the preferred stimulant for adults with ADHD [6]. Ultimately, we decided to trial LDX 30 mg QAM, which has the added benefit of once-daily dosing and less abuse potential than MAS IR, as well as a smoother onset and offset of action than MAS XR [7].

Over the course of two months, she reported LDX 30 mg QAM as ineffective. After confirming medication adherence, timing relative to symptoms, effects of acidic foods, and any other new or over-the-counter medications, we decided to titrate the dose to 60 mg in order to produce a discernible clinical change and to leave room for upward or downward titration. Soon after, our patient reported jitteriness and tachycardia on 60 mg. Noting that stimulants have individual therapeutic windows [8], that the adverse effects were currently exceeding the benefit, that cardiovascular safety must be prioritized, and that we should have more room to taper her downward if needed, we made the decision to taper her down to LDX 50 mg QAM. At the 50 mg dose, our patient reported no side effects and adequate symptom reduction.

However, soon after, due to the stimulant shortage, LDX 50 mg became unavailable in any dosage formulation, adding up to 50 mg cumulatively. When deciding on a new trial, we chose MAS IR 20 mg BID, as our patient had documented prior success on MAS IR 20 mg BID; it is within the same class as LDX, and we are able to approximate the LDX coverage profile with BID MAS IR dosing [9]. Although our patient had been on this medication previously, she reported it was ineffective, noting symptoms of inattention and executive dysfunction. Additionally, it was increasingly difficult to obtain due to the shortage.

We speculate that the reasoning behind the ineffectiveness at this trial may have been due to either a manufacturer discrepancy or our patient responding better to D-AMPH than levoamphetamine (L-AMPH), since she responded well to LDX, and LDX is a prodrug that, after ingestion, releases only D-AMPH over the course of 10-12 hours [10]. D-AMPH IR, also known as Zenzedi, ProCentra, or Dexedrine, is FDA-approved for the treatment of ADHD in ages 6 and above, and its use is much less frequent than MAS IR. Therefore, our clinic switched her to D-AMPH IR 20 mg BID and then tapered down to D-AMPH IR 20 mg QAM due to our patient reporting that BID dosing was excessively strong and provided intense focus for longer than necessary. Since early 2024, our patient has been stable on D-AMPH IR 20 mg QAM and has maintained adequate symptom reduction.

The main difference between MAS and D-AMPH lies in the amphetamine salt concentrations. MAS contains a 3:1 ratio of D-AMPH to L-AMPH, while D-AMPH contains only D-AMPH [11]. The prescription rates of LDX, also known as Vyvanse, have significantly increased over the past decade, mostly due to its lower potential for abuse from its mechanism of action than any other long- or short-acting amphetamine formulations [12]. There also lies a difference in the efficacy and mechanism of action of D-AMPH and L-AMPH. Both molecules are amphetamines; however, levoamphetamine is more effective for hyperactivity and aggression than attention deficit, and the dextroamphetamine isomer is equally effective for all three symptoms [13]. The pharmacologic difference between the isomers remains that dextroamphetamine is more potent dopaminergically than levoamphetamine and that levoamphetamine is more potent noradrenergically than dopaminergically [14].

Because of this, psychiatric prescribers have generally viewed IR D-AMPH as having a higher potential for abuse than IR MAS, and this is likely a contributing factor to the declining prescription rates of IR D-AMPH. However, there are no data showing a comparison of abuse liability between IR D-AMPH and IR MAS. The only data comparing the two isomers in terms of central dopaminergic effects and self-reinforcement is a 1973 experiment, which found D-AMPH to be more reinforcing than L-AMPH in rats. Despite this, it is unknown if this would translate to human subjects at clinical doses [15].

Although the argument could be made that LDX is one of the most abuse-resistant stimulant formulations and that it releases only dextroamphetamine, it is also important to note that the release mechanism of LDX prevents individuals from consuming the drug via any other method of administration than orally, as it is a prodrug. In all, IR D-AMPH may be a viable alternative to IR MAS amid the stimulant shortage; however, more research must be conducted before this can be concluded, as our sample size consisted of only one patient.

## Conclusions

The stimulant shortage has significantly limited the medication options for treating ADHD. In this case, we successfully switched the medication of an affected patient despite limited options. We demonstrated that dextroamphetamine sulfate may be a viable option to substitute other amphetamine-based medications and specifically MAS IR during the shortage, as it is comparable in efficacy and pharmacology. However, more research is needed before a conclusion can be drawn about the practical viability of this switch, specifically focusing on abuse potential and head-to-head experiments comparing D-AMPH to MAS, as our clinic evaluated this treatment option in only one patient.
